# The Association of Surgeons

**Published:** 1920-01-24

**Authors:** 


### The Hospital

The Workers' Newspaper of Administrative Medicine and Institutional Life, Administration, National Insurance and Health.

No. 1755, Vol. LXVII. SATURDAY, JANUARY 24, 1920. PRICE TWOPENCE

### THE ASSOCIATION OF SURGEONS.

The phase of social evolution through which we are now passing offers many instances of both professional and commercial appreciation of the process of bringing to bear at a common centre results of effort formerly distributed throughout the country. If illustration of the success attending the centralisation be needed, one has not far to turn, for on every hand we see the financial success of skilfully concerted endeavour, and it is a matter for great satisfaction when, on any occasion, one can point to a corresponding tendency in strictly scientific circles.

Not that this has been absent in the past. In the realms of pure science, the recent war, which was a conflict essentially swayed by scientific invention, brought out the urgent necessity and the perfected workings of many such schemes, and similarly indeed, thanks to a greatly renovated and very changed R.A.M.C. organisation, was administrative medical organisation in the field developed to an almost undreamed of extent. Scarcely was armistice signed before the fruits of these lessons began to show in civilian work at home, and already, one after the other, many of the stumbling blocks and hindrances to scientific medical advance have been swept aside. Now we welcome the latest development in this train of circumstance and outline some of the lasting effects likely to accrue therefrom.

The new Association of Surgeons, at the inaugural meeting of which Sir Rickman Godlee recently presided, is described as the direct outcome of a meeting held just before the war at the suggestion of Sir Berkeley Moynihan. About forty representative men were present at the Royal College of Surgeons on May 26, 1914, and a committee was then appointed to draw up rules, to circularise the members of the surgical staffs of hospitals connected with teaching schools, and to summon a meeting of all those who agreed to join the association. Now, after an interval including some of the saddest but most eventful and instructive years in history, this meeting has taken place, and meanwhile the committee has framed its rules, guided by much invaluable experience.

The essential requirement was first a representative union of the three great surgical schools of the British Isles. Hitherto British surgery has had no common home; each man has been more or less instinctively attached to the place of his early training. Multiplicity of possible degrees and their relative values are subjects open to considerable discussion, but, though it is true that the obtaining of the F.R.C.S.Eng. calls for an intellectual display hardly equalled by any other medical qualification, yet we doubt whether the fact that degrees and diplomas do not make a surgeon has been sufficiently recognised. On this and similar points has often arisen a rivalry, or at least a lack of understanding, almost as vital in its power for ill as our insular habits of the past, and the meetings of the new Association should do much to repair the unity within.

The question of age limitation and number of membership we may pass, but, in view of the circumstances typical of the average meeting of a medical society, the second part of the Committee's suggestions are interesting and suggestive of that unhindered progressive spirit we so badly require in medical circles. In the first place, as we see it concisely described, the discussions are to be, in every meaning of the word, free, and the expression of thought will be untrammelled by the dread, which even the least prudent must feel, lest what he might say confidentially to his friends should be repeated in a garbled form from the housetop.

Therefore the first communications will probably be short and made *viva voce*. Probably, also, no reporters will be present and no report allowed to be sent to the journals and newspapers, although Fellows may later publish as they will: a great loss, perhaps, to the current Press, but nevertheless we must admit it as a justifiable provision under modern conditions. Officially the *British Journal of Surgery* is suggested as the most appropriate channel by which to bring the considered results before the profession as a whole, and, while applauding this choice, we may say we look forward to reading in those pages the valued and urgently-needed results of a centralisation of all that is best in British surgery—a section of the country's economy far too vital to be left still in any form of division against itself.

